# Quality of Life and Needs in Caregivers: Results From the Prospective Multicentric Open-Label Randomized Study of Informal Caregivers of Elderly Patients

**DOI:** 10.3389/ijph.2023.1605459

**Published:** 2023-08-30

**Authors:** Astrid Pozet, Sophie Darnis, Magalie Bonnet, Aurélia Meurisse, Tienhan Sandrine Dabakuyo-Yonli, Catherine Lejeune, Philippe Fagnoni, Maryse Gaimard, Patrick Manckoundia, Clémence Quibel, Mélanie Marchand, Amélie Anota, Virginie Nerich

**Affiliations:** ^1^ Methodological and Quality of Life Unit in Oncology, Centre Hospitalier Universitaire de Besançon, Besançon, France; ^2^ UMR1098, Interactions Hôte-Greffon-Tumeur/Ingénierie Cellulaire et Génique, INSERM, Université de Bourgogne-Franche-Comté, Etablissement Français du Sang Bourgogne-Franche-Comté, Besançon, France; ^3^ Department of Clinical Research and Innovation, Centre Léon Bérard, Lyon, France; ^4^ Department of Psychology, EA 3188, UFR Sciences du Langage de l'Homme et de la Société, Université de Bourgogne-Franche-Comté, Besançon, France; ^5^ Epidemiology and Quality of Life Research Unit, INSERM U1231, Georges François Leclerc Centre - UNICANCER, Dijon, France; ^6^ The French National Platform Quality of Life and Cancer, Dijon, France; ^7^ INSERM, CIC1432, Clinical Epidemiology Unit, Centre Hospitalier Universitaire de Dijon, Dijon, France; ^8^ Department of Pharmacy, Centre Hospitalier Universitaire de Dijon, Dijon, France; ^9^ INSERM U866, Centre Hospitalier Universitaire de Dijon, Dijon, France; ^10^ Laboratoire Interdisciplinaire de Recherche Sociétés, Sensibilités, Soin (LIR3S), UMR 7366, Université de Bourgogne-Franche-Comté, Dijon, France; ^11^ Department of Gerontology, Hôpital de Champmaillot, Centre Hospitalier Universitaire de Dijon, Dijon, France; ^12^ Pôle de Gérontologie et d'Innovation (PGI) de Bourgogne-Franche-Comté, Besançon, France; ^13^ Department of Human and Social Sciences, Centre Léon Bérard, Lyon, France; ^14^ Department of Pharmacy, Centre Hospitalier Universitaire de Besançon, Besançon, France

**Keywords:** anxiety, burden, depression, quality of life, caregiver, elderly, intervention

## Abstract

**Objectives:** To assess health-related quality of life (QoL) in caregivers of elderly patients with chronic disabilities receiving, or not receiving, social worker support.

**Methods:** This multicenter open-label randomized study assigned caregivers to receive an information booklet, exclusively, or with social worker support. Caregivers completed Short Form-36 (SF-36) and Hospital Anxiety Depression Scale quarterly, and Zarit Burden Interview each semester, for 24 months. We reported caregiver QoL mean changes at 12 and 24 months (M12, M24). Longitudinal QoL analysis up to M24 used mixed models for repeated measures (MMRM).

**Results:** Among the 179 caregivers randomized from 2015 to 2019, the SF-36 physical and mental component summary showed no significant changes at M12 and M24, in terms of neither anxiety nor burden. However, depression significantly increased (M12: 1.4 ± 4.0; M24: 1.7 ± 4.1) with significant adjusted mean increase using MMRM at M24: 3.4 [0.6–2.5] in the control group, exclusively.

**Conclusion:** These findings call for better recognition of the social support to prevent caregiver QoL deterioration and alleviate their depression early in the course of the disease.

**Clinical Trial Registration:** ClinicalTrials.gov, identifier NCT02626377.

## Introduction

During recent decades, life expectancy in technologically-advanced countries has continuously increased*,* and a higher prevalence of chronic diseases has become a major public-health policy concern [1]. Health systems at different levels of health and social care need to coordinate their efforts to provide appropriate services in daily living for older people with functional limitations. Public support for informal care is one of the most important public policy measures for the future sustainability of health and social care in ageing populations [2, 3].

In Europe, caregiver incidence is rising steadily, and forecasts indicate that 25% of the people in employment will also assume caregiving responsibilities in 2030. Last updated estimates in France reported 9.3 million (14.8%) caregivers in 2021, including 4.3 million of caregivers of the elderly [4]. Informal caregivers usually include spouse, parents, adult children, friends, or neighbors providing any type of unpaid assistance with activities of daily living, regardless of the duration of daily time devoted to caregiving and accompanying [5]. Although the estimated number of caregivers varies between countries, depending on informal care definition and measurement, many European countries considered that almost 20% of the people aged 50 years or older were informal caregivers in 2017 [6]. This caregiving role is physically, emotionally, socially, and financially demanding, and about one third of the caregivers have admitted that they experience negative effects in their familial and social life, that they lack time to pursue leisure activities, and that their psychological and physical health may be greatly affected [7]. In 2017, the *European Pillar of social rights* makes explicit commitments to caregivers, including their right to flexible working and access to care services, and initiatives to support and preserve their employment, health, and wellbeing [8–10].

Caregivers may be supported through different interventions, ideally integrated into usual management. Recent approaches showed that home-based, nurse-led-interventions for caregivers incorporated into primary care improve health-related quality of life (QoL) in caregivers of patients with chronic or disabling conditions [11]. Whereas support to caregivers is integrated into early palliative care in oncology [12], other helpful contributions may be provided by community resources or support groups [13]. Social workers, involved in the management of older people, and experimenting in facilitating the caregiving role, can also be considered as privileged partners not only for patients but also for the specific population of caregivers, which suggests addressing the scheme provided through the ICE study. In addition, possible deployment using regional and nationwide networks is a tremendous asset and a promising and economically viable option.

In order to promote caregiver support and wellbeing, careful monitoring of caregiver QoL needs to be performed [14, 15]. Obviously, caregiver QoL is closely linked to the health status of the helped patient, and profound changes in caregiver lifestyle are regularly required, with practical, organizational, and potentially economic problems associated with them, according to the patient health status [16]. From initial diagnosis, through successive phases of stabilization, remission, or progressive decline and palliation, exposure to chronic stress may directly affect caregiver mental and physical health, and appropriate caregiver QoL assessment with access to support and respite is essential to prevent caregiving exhaustion. As part of the overall concept of QoL, monitoring caregiver anxiety and depression symptoms and burden is of particular importance, and their related scores are useful to detect early signs of mental health deterioration [17–21]. These scores are used in caregivers to further preserve their health, or to prevent health deterioration, which could subsequently jeopardize their ability to care for themselves and for the patient. Caregiver needs further require appropriate characterization to provide dedicated support with efficient, appropriate, and timely services including psychosocial services, respite, training, education, and long-term care. In addition, better identification of the caregivers the most in need is also necessary to relieve and ideally prevent burden associated with caring [17, 18].

This French prospective multicentric cohort of the *Informal Carers of Elderly* (ICE) study was initiated in 2015 in the Burgundy-Franche-Comté region (2.8 million inhabitants, 4.4% of the French population) [22], and included i) an observational study planned to enroll 7,604 caregivers of patients aged 60 years and older recently diagnosed with chronic disease for a 5 year period, with the primary endpoint defined as caregiver QoL assessment over 5 years; ii) a randomized trial focusing on the first 2 years of caregiving, to compare caregiver QoL at 1 year between caregivers receiving an information booklet alone and those receiving social worker support, according to the patient disease. It is necessary to demonstrate whether the social worker intervention benefits caregivers. The trial faced significant recruitment difficulties. Although several amendments were adopted to overcome barriers in recruitment and revision of the initial objectives, low accrual prompted the steering committee to stop enrollment in May 2019. The present work aims to report caregiver and patient characteristics and assess caregiver QoL, anxiety and depression, and burden at one and 2 years, as well as changes over the 2-year period in caregivers receiving an information booklet combined or not with support from social workers.

## Methods

### Design and Study Population

Physicians received patients diagnosed with one of the specified diseases and detected the potential primary caregiver during consultation. Caregivers were ≥18 years, identified by the patient or self-identified as primary caregiver, not employed by a healthcare organization, residing in the French region of Burgundy-Franche-Comté. Caregivers supported patients aged ≥60 years with a neuro-degenerative disease (idiopathic Parkinson’s or Alzheimer disease), cancer (breast, prostate, or colorectal), age-related macular degeneration, or neuro-vascular disease (stroke). Caregivers of patients living in institution, and caregivers under legal protection were not included. Inclusion/exclusion criteria were previously detailed [23].

A clinical research associate met the caregiver to gather all required information regarding the study (information sheet, informed consent), enrolled the caregiver, and indicated the allocation group. More details were previously reported [23]. All caregivers provided written informed consent.

Faced with low recruitment to achieve the initially planned sample size, and despite several amendments, the initial goal was unachievable, and the final sample size of the cohort ICE did not allow the statistical analyses initially planned [23] and specifically the lack of power did not allow a direct comparison of the supportive intervention and control groups.

### Intervention Description

Caregivers were randomly assigned (1:1 ratio) to receive an information booklet and intervention from a social worker in the supportive intervention group (SIG) or to receive exclusively an information booklet in the control group (CG). Randomization was done by the data manager with an interactive web response system, using a minimization technique with stratification according to center, age (80 years or older versus below 80 years), gender, and stage (severity of the disease). Investigators and caregivers were not masked to group allocation.

The theoretical framework justifying social worker intervention is to prevent caregiver QoL deterioration and to better support their involvement and communication, thereby contributing to preserving the quality of care provided to the patient. Social worker intervention used the Linear Analogue Scale Assessment (LASA) questionnaire and semi-directive interviews to support the emergence of caregiver needs and specifically address their needs through counselling regarding home services, medical home care, community services (support group), proposing services to promote safety and assist in daily needs (meal delivery, medical alert service), counselling from a psychologist, admission of caregivers for respite care, and encouraging caregivers to take care of themselves and regularly attend consultations with their physician. Interventions from social workers were scheduled at 6, 12, 18, and 24 months (M6, M12, M18, and M24) from inclusion and consisted of a 1 hour visit at the caregiver’s home with the intention of evaluating the level of difficulties experienced by the caregiver using the LASA questionnaire [24], assessing caregiver needs, and detecting early signs of burden through a standardized semi-structured interview (Table 1). Booklets provided access to relevant external assistance structures, support programs, and included information regarding local legislation, administrative procedures, daily living management, and potential consequences related to the caregiving role.

**TABLE 1:** Social worker intervention, based on the linear analogue self-assessment questionnaire and semi-structured interview performed at caregiver home (Informal Carers of Elderly study, France, 2015–2019).

Main domains	Description
One-hour semester visit performed at caregiver home	
➢Linear analogue self-assessment questionnaire completion [24]	Assessment of global quality of life, mental, physical wellbeing, fatigue.
➢Semi-structured interview	
Awareness of the commitment as a carer	**Questions asked at the first visit:** What is your relationship with the person you are helping? How long have you been providing assistance?
	-How do you organise the help for your relative?
Caregiver/patient relationship	-How would you describe your relationship with your relative since disease diagnosis?
	-What does your role as a carer provide to you?
	**Questions asked on the 2nd, 3rd and 4th visits:**
	-Do you feel that you are managing to cope with your relative’s illness? What are the disorders or symptoms of the disease that you find most difficult to manage on a daily basis? What are the main difficulties you encounter?
	-Do you consider yourself a caregiver? How has your relationship with your loved one changed since the diagnosis was announced?
Implications and consequences on your personal life	- How are you coping with your relative’s illness?
	- What are the main repercussions of this caregiving role on your life? Does your caring role affect your health, your financial situation, your professional activity?
Expectations and needs as a carer	- Do you have expectations and needs as a caregiver?
	- Can you rely on someone for leisure time when needed?
	- Do you have any spare time?
	- Do you use home or caregiver services?
	- Do you feel you have the necessary information or know where to find it?
**Follow-up**	
Action plan at the social worker discretion, using linear analogue self-assessment questionnaire, and semi-structured interview	-Identification of caregiver needs and detection of early signs of burden based on the collected responses form the interview
	-Provide appropriate accompaniment and support (offer valuable information for support in everyday life and home services, i.e., outside therapeutic counselling, or training to care. Main trends included medical home cares, services to promote safety and assist in daily needs (meal delivery, medical alert service), counselling from psychologist, community services (support group); Social workers globally encourage caregivers to take care of themselves and to regularly consult their physician for themselves; referral to appropriate structures of care depending on caregiver situation (admission for respite care for caregivers or caregiver/patient dyads)
	The decision for the most adequate solution to provide was at social worker discretion.

### Variable and Instruments

Besides patient demographics (gender, age, disease), caregiver baseline characteristics mainly included: gender, age, marital status, patient-caregiver relationship, professional activity, profession and incomes, impact from caregiving on professional and financial situation, professional help requested, involvement (daily activities (grooming/dressing…); domestic chores (cleaning, grocery shopping, meals…); Administrative management (accounting, mails, decisions); Medical support (accompaniment to medical appointments, medical cares); Physical support services; Financial assistance; Moral and emotional support; Medical decision support.

Caregiver questionnaires were self-completed at home using paper-pencil or electronically assessed through a secure web platform according to their preferences. Questionnaires were sent to caregivers, regardless of the social worker intervention, in order to prevent any bias of desirability in the SIG.

The SF-36 questionnaire chosen to assess QoL is the most frequently used generic instrument translated and validated in French for a wide range of diseases [19, 25], providing quick answers on specific issues (5–10 min for full completion) and generating physical component summary (PCS) and mental component summary (MCS) scores, as well as scores per dimension (physical functioning, role physical, bodily pain, mental health, emotional role, social functioning, vitality, general health, and health transition). The Hospital Anxiety Depression Scale (HADS) questionnaire used to assess anxiety and depression in different pathologies, in hospitalized and non-hospitalized patients, and also in apparently healthy persons, was validated in French [20, 26]. The Zarit Caregiver Burden Interview (ZBI), also validated in French, has been reported as a reliable tool for the assessment of caregiver burden, i.e., degree of exhaustion or psychological fatigue in caregivers [21].

All questionnaires were self-administrated quarterly except for ZBI administrated each semester. Detailed procedures have been previously reported [23].

Scores for SF-36 subscales were computed on a 0 (worse QoL) to 100 (best QoL) point scale [27]. The minimal important difference (MID) was defined as the smallest change on any scale within an individual or at the group level. SF-36 MID was fixed at 5 points [28]. HADS reported a raw score from 0 (absence of trouble) to 21 (severe trouble) points [20], with an MID for HADS anxiety score fixed at 1.32 points, and for depression a score of 1.4 points [29]. The ZBI reported a raw score from 0 (no burden) to 88 (severe burden) points [21], with MID defined as half of the standard deviation observed at baseline, as usually performed for scores with no previously determined MID. Of note, increased SF-36 scores translated to an improved status, and increased HADS and ZBI a decreased status.

### Endpoints

The primary endpoint was QoL SF-36 physical component summary (PCS) and mental component summary (MCS) score changes at M12 and M24 compared to baseline in each group. Secondary endpoints included i) QoL changes in SF-36 subscales, anxiety/depression, and burden at M12 and M24 compared to baseline; ii) longitudinal assessments of caregiver QoL, anxiety/depression, and burden over the 2 years. Exploratory analyses were performed to assess changes from baseline in caregiver involvement in activities of daily living, and to assess professional and financial support requested by caregivers, at M12 and at M24.

### Statistical Analyses

Analyses included all caregivers randomized, with the completed questionnaire at baseline, and all data available were considered at each timepoint. Only descriptive analyses per group were conducted.

Socio-demographic, clinical patient characteristics, and caregiver characteristics were described in the global population and in each group. Categorical variables were expressed using number and frequency (n,%), and continuous variables used median (min-max). Mean (standard deviation)/median (min-max) scores were described at each timepoint in both groups. No statistical comparisons between randomization groups were done due to the limited sample size.

Reliability of the questionnaires was evaluated with the assessment of the internal consistency using a Cronbach’s alpha coefficient for each dimension of the SF-36, HADS, and ZBI at baseline. A Cronbach’s alpha of at least 0.70 was expected [30, 31].

To assess potential attrition bias, baseline characteristics of caregivers with early discontinuation at M12 and at M24 were compared to those continuing the study. *p*-values were provided to help in interpreting the results. Quantitative data were compared using a Student’s t-test. Categorical data were compared using a Chi-square test.

Mean changes in SF-36, HADS, and ZBI scores from baseline to M12 and M24 are presented per group with a paired *t*-test fixed at 5% for statistical significance.

Mixed models for repeated measures were used for longitudinal analysis including all timepoints up to M24 and including the following effects: randomization group, time, allocation-by-time interaction, adjustment on baseline score, and baseline score-by-time interaction. Random effects on intercept and time were used in order to reflect individual variations. Adjusted mean changes at M12 and M24 were reported with 95% confidence interval for SF-36-PCS and MCS scores, HADS anxiety and depression, and burden, per group. Statistical and MID clinical significances were indicated.

Analyses were performed using SAS version 9.4 software (SAS Institute Inc., Cary, NC, USA).

## Results

**FIGURE 1:**
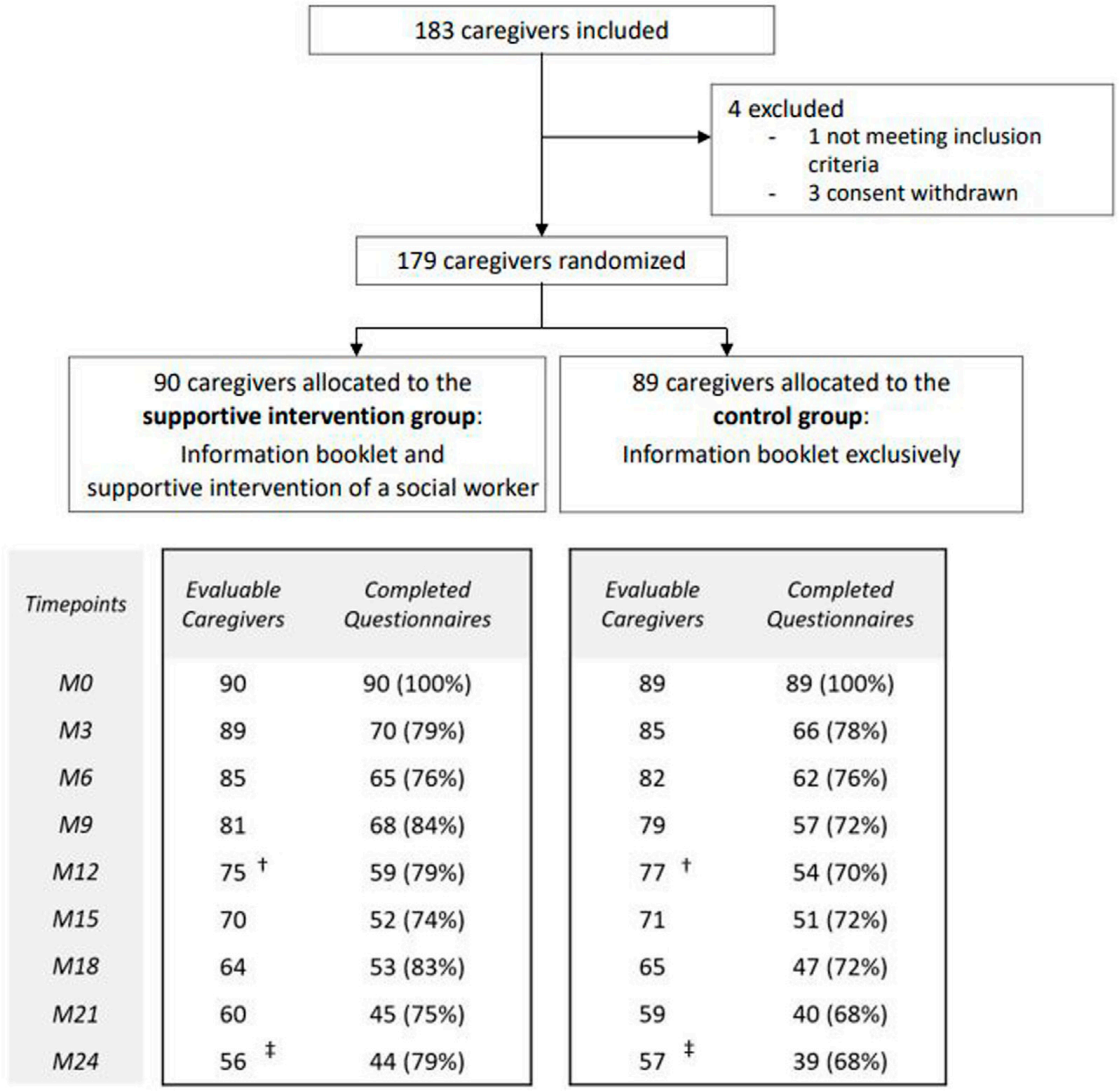
Trial profile (Informal Carers of Elderly study, France, 2015–2019). Note: Only questionnaires with at least one scored dimension were considered for analysis. ^†^Study discontinuation at Month 12 (M12) (Supportive intervention group: *n* = 15 [caregiver decision, *n* = 12, patient death, *n* = 3]; Control group: *n* = 12 [caregiver decision, *n* = 8, patient death, *n* = 4]).^‡^Study discontinuation at Month 24 (M24) (Supportive intervention group: *n* = 34 [caregiver decision, *n* = 23; patient death, *n* = 9; relocation in institution, *n* = 1 outside the region, *n* = 1]; Control group: *n* = 32 [caregiver decision, *n* = 20; patient death, *n* = 10; caregiver death, *n* = 1; relocation in institution, *n* = 1]).

From October 2015 to May 2019, 183 caregivers were recruited and 179 were randomly assigned in the SIG (*n* = 90) or in the CG (*n* = 89). Each caregiver completed questionnaires at inclusion and the completion rates reached reliable percentages (mainly >70%) for each follow-up timepoint. In the SIG, the rates were 59/75 (79%) at M12, and 44/56 (79%) at M24, and in the CG, 54/77 (70%) at M12, and 39/57 (68%) at M24. Details including reasons for early study discontinuation are presented in Figure 1.

### Caregiver Characteristics at Baseline

Caregivers’ median age was 65 (29–92) years, and more caregivers were women (*n* = 120, 67%) identified as spouse (*n* = 118, 66%), or as child helping mother/father (*n* = 42, 23%). The related patient population had a median age of 73 (60–94) years, including 92 (52%) patients with cancer, 66 (37%) with neurodegenerative disease, 11 (6%) with age-related macular degeneration, and 10 (5%) with stroke. Most of the caregivers were retired, however 42 (22%) caregivers still had a professional activity, among them 15 caregivers declared that supportive care had an impact on their professional life and required time arrangement (*n* = 10). Twenty-three (15%) caregivers declared average household incomes below €1500 per month. At inclusion, 5 (3%) asked for financial support and 25 (14%) for professional support (Table 2). At baseline, most of the caregivers were involved in five main support areas: moral and emotional support (*n* = 172, 96%), medical decision support (*n* = 172, 96%), medical support (*n* = 136, 76%), domestic chores (*n* = 120, 67%), administrative management (*n* = 119, 66%). Half of the caregivers declared to provide financial assistance (*n* = 94, 53%) (Table 2).

**TABLE 2:** Patient and caregiver characteristics in all randomized caregivers, and in the supportive intervention group and in the control group (Informal Carers of Elderly study, France, 2015–2019).

	SIG (*n* = 90)	CG (*n* = 89)	All randomized caregivers (*n* = 179)
Patients			
Gender			
Male	39 (43)	40 (45)	79 (44)
Female	51 (57)	49 (55)	100 (56)
Age	73 (60–94)	73 (60–94)	73 (60–94)
Disease			
Cancer	46 (51)	46 (51)	92 (52)
Cancer localization			
Colorectal cancer	11 (12)	10 (10)	21 (12)
Prostate cancer	12 (13)	13 (15)	25 (14)
Breast cancer	23 (26)	23 (26)	46 (26)
Cancer severity			
Non metastatic/adjuvant	27 (59)	27 (60)	54 (60)
Metastatic/advanced	19 (41)	18 (40)	37 (40)
Not specified	0	1	1
Neuro-degenerative disease	33 (37	33 (37)	66 (37)
Type of neuro-degenerative disease			
Alzheimer disease	23 (26)	23 (26)	46 (26)
Idiopathic Parkinson’s disease	10 (11)	10 (11)	20 (11)
Alzheimer severity			
Low: MMSE≥26	2 (9)	0	2 (4)
Mild: 20≤MMSE<26	12 (52)	13 (57)	25 (55)
Moderate: 10≤MMSE<20	8 (35)	10 (43)	18 (39)
Severe: MMSE<10	1 (4)	0	1 (2)
Idiopathic Parkinson’s disease severity			
Stade I	1 (10)	2 (20)	3 (15)
Stade II	4 (40)	4 (40)	8 (40)
Stade III	4 (40)	2 (20)	6 (30)
Stade IV	1 (10)	2 (20)	3 (15)
AMD	6 (6)	5 (6)	11 (6)
AMD severity			
Retrofoveolar exudative	2 (50)	4 (80)	6 (67)
Extrafoveolar exudative	2 (50)	1 (20)	3 (33)
Not specified	2	0	2
Stroke	5 (6)	5 (6)	10 (5)
Stroke severity			
Barthel score	95 (40–100)	100 (65–100)	100 (40–100)
Rankin score	1 (0–5)	1 (0–4)	1 (0–5)
Caregivers			
Gender			
Male	33 (37)	26 (29)	59 (33)
Female	57 (63)	63 (71)	120 (67)
Age	67 (29–92)	65 (34–92)	65 (29–92)
Marital status/living situation			
Married, common-law couple, couple	71 (79)	79 (89)	150 (84)
Other (single, separated, divorced, or widowed)	19 (21)	10 (11)	29 (16)
Caregiver-patient relationship (caregiver taking care of his/her)			
Spouse	59 (66)	59 (66)	118 (66)
Mother/Father	20 (22)	22 (25)	42 (23)
Other family members (sister, brother, mother/father-in-law, uncle/aunt, grandmother)	5 (5)	5 (6)	10(6)
Other (friend, neighbor, ex-husband)	6 (7)	3 (3)	9 (5)
Professional situation			
Professional activity	22 (24)	20 (22)	42 (22)
Caregiving impact on caregiver professional life			
Yes	7 (32)	8 (40)	15 (36)
No	15 (68)	12 (60)	27 (64)
Type of impact on caregiver professional life			
Work-time arrangements	5 (63)	5 (71)	10 (63)
Other	2 (37)	3 (29)	5 (31)
Retired	62 (69)	61 (69)	123 (69)
Other (sick leave, unemployment, job training)	6 (7)	8 (9)	14 (9)
Past or current professional occupation			
Farmer	0	3 (4)	3 (2)
Craftsman, shopkeeper, business owner	6 (7)	2 (2)	8 (5)
Executive, intellectual profession	22 (25)	18 (22)	40 (24)
Middle level profession	9 (10)	7 (9)	16 (9)
Employee	46 (53)	45 (56)	91 (54)
Worker	3 (3)	6 (7)	9 (5)
Other (without profession, job training or student)	2 (2)	0	2 (1)
Not specified	2	8	10
Household incomes €/month			
< €800	3 (4)	3 (4)	6 (4)
From €800 to €1,500	8 (10)	9 (11)	17 (11)
From €1,501 to €3,000	45 (56)	41 (52)	86 (54)
≥ €3,001	24 (30)	26 (33)	50 (31)
Not specified	10	10	20
Caregiving impact on caregiver financial situation	5 (6)	4 (4)	9 (5)
Financial help requested by the caregiver	3 (3)	2 (2)	5 (3)
Professional help requested by the caregiver	13 (14)	12 (13)	25 (14)
Involvement in patient activities			
Daily living activities	8 (9)	10 (11)	18 (16)
Domestic chores	59 (66)	61 (68)	120 (67)
Administrative management	65 (72)	54 (61)	119 (66)
Medical support	69 (77)	67 (75)	136 (76)
Physical support services	29 (32)	27 (30)	56 (31)
Financial assistance	49 (54)	45 (48)	94 (53)
Moral and emotional support	86 (96)	86 (97)	172 (96)
Medical decision support	71 (79)	71 (80)	142 (79)

Note: SIG, supportive intervention group; CG, control group; AMD, age-related macular degeneration; MMSE, mini mental state examination. Daily living activities (grooming/dressing, etc.); Domestic chores (cleaning, grocery shopping, meals, etc.); Administrative management (accounting, mails, decisions); Medical support (accompaniment to medical appointments, medical cares). Data are median (range) or n (%).

Reliability of the questionnaire was reached with a Cronbach’s alpha coefficient of at least 0.70 for each dimension (ranging from 0.79 to 0.90 for each dimension).

Caregivers with early discontinuation at M12 and at M24 showed more unfavorable characteristics at inclusion (Supplementary Material S1).

### Health-Related Quality of Life

In SF-36-PCS and -MCS mean scores, no clinically significant decreases were observed at M12 or at M24 in each group. However, a statistical difference for PCS at M12 was noted in each group (Table 3).

**TABLE 3:** Short Form-36, Hospital Anxiety Depression Scale and Zarit Burden Interview, mean scores at baseline and mean score differences at months 12 and 24 in the supportive intervention group and in the control group (Informal Carers of Elderly study, France, 2015–2019).

	Allocation group	Score at baseline		Score difference			
		n	Mean (SD)	M12 – baseline		M24 – baseline	
				*n*	Mean (SD)	*n*	Mean (SD)
Short Form-36							
Physical Component Summary	SIG	90	47.2 (8.7)	59	−4.1 (8.2)*	43	−2.4 (6.9)
	CG	89	49.6 (8.8)	53	−4.2 (8.4)*	38	−3.6 (8.0)
Mental Component Summary	SIG	90	44.9 (10.3)	59	−0.3 (11.5)	43	0.1 (10.8)
	CG	89	44.9 (11.1)	53	−1.0 (9.9)	38	0.5 (10.9)
Short Form-36 subscales							
Physical Functioning	SIG	90	85.9 (18.9)	59	−10.2 (17.2)*	44	−8.5 (16.5)*
	CG	89	85.5 (18.0)	54	−9.9 (19.1)*	38	−9.0 (18.1)*
Role Physical	SIG	90	69.4 (38.2)	59	−11.4 (38.4)*	44	**−8.0 (39.2)**
	CG	89	75.0 (37.9)	54	**−14.3 (40.5**)*	39	**−7.9 (41.1)**
Bodily Pain	SIG	90	53.9 (34.1)	59	**−7.1 (28.1)**	44	−0.5 (20.9)
	CG	89	68.1 (32.2)	54	−11.1 (33.2)*	39	−11.3 (33.3)*
Mental Health	SIG	90	62.7 (17.5)	59	−2.4 (17.0)	44	−0.2 (15.8)
	CG	89	62.9 (20.5)	54	−3.2 (15.6)	39	−1.4 (17.7)
Role Emotional	SIG	90	67.4 (38.7)	59	−1.1 (45.0)	43	−3.1 (45.3)
	CG	89	69.7 (40.7)	53	−3.1 (37.7)	39	−3.4 (45.8)
Social Functioning	SIG	90	78.2 (21.8)	59	−7.4 (25.0)*	44	−4.0 (22.7)
	CG	89	78.5 (21.1)	54	−9.9 (24.1)*	39	**−5.8 (21.6)**
Vitality	SIG	90	55.4 (17.9)	59	−3.0 (16.3)	44	−2.5 (17.0)
	CG	89	59.3 (20.5)	54	−5.4 (15.2)*	39	−3.5 (17.7)
General Health	SIG	90	62.9 (16.4)	59	−3.0 (13.5)	44	−1.3 (13.4)
	CG	89	63.9 (19.0)	54	−1.8 (11.4)	38	−1.2 (12.9)
Health Transition	SIG	90	48.0 (15.5)	58	1.3 (21.2)	44	−1.7 (19.7)
	CG	89	50.3 (17.5)	53	−1.9 (18.2)	39	**5.8 (18.6)**
Hospital Anxiety Depression Scale							
Anxiety	SIG	90	7.6 (4.0)	59	−0.1 (3.2)	43	−0.4 (3.1)
	CG	89	7.9 (4.3)	54	0.3 (3.9)	39	−0.4 (3.2)
Depression	SIG	90	4.3 (3.4)	59	0.6 (3.9)	43	0.9 (3.4)
	CG	89	4.3 (3.6)	54	1.4 (4.0)*	39	1.7 (4.1)*
Zarit Burden Interview							
Burden	SIG	87	19.7 (13.7)	54	2.0 (10.5)	44	1.1 (13.2)
	CG	88	17.7 (15.1)	53	6.4 (11.4)*	38	6.7 (15.7)*

Note: M12, M24: Month 12, 24; SIG: supportive intervention group; CG: control group; SD: standard deviation; Number of questionnaires completed for the considered item at each timepoint are presented (n). Minimal important differences (MID) were fixed at 5 points for Short Form-36 scores, 1.32 points for Hospital Anxiety Depression Scale anxiety score, 1.4 points for Hospital Anxiety Depression Scale depression score, and 7.2 points for Zarit Burden Interview score. In bold: MID significant.

*Paired *t*-test *p* < 0.05.

Regarding the SF-36 subscales, the CG showed a clinically and statistically significant decrease in the mean score for physical functioning and bodily pain at M12 and M24, physical role, social functioning, and vitality at M12. In the SIG, a significant decrease in the mean scores in physical functioning were reported at M12 and M24, and in physical role and social functioning at M12 (Table 3).

### Anxiety and Depression

While no difference in anxiety mean score was reported, depression mean score was clinically and statistically increased in the CG at M12 and M24. No differences in anxiety and depression mean scores were reported in the SIG regardless of each timepoint (Table 3).

### Burden

Mean ZBI score was not clinically different from baseline to each timepoint in each group. Of note, a statistically significant increased ZBI score at M12 and M24 was observed in the CG (Table 3).

### Longitudinal Analysis

The mixed models for repeated measures did not show clinically significant differences at M12 or at M24 for SF-36, HADS anxiety, and ZBI (Table 4). However, a significant change in HADS depression score at M24 was identified in the CG (adjusted mean change 3.4 [0.6–2.5]).

**TABLE 4:** Caregivers adjusted mean change over time (mixed model for repeated measures) in Short Form-36 physical component summary, mental component summary, in Hospital Anxiety Depression Scale, and Zarit Burden Interview in each allocation group (Informal Carers of Elderly study, France, 2015–2019).

	SIG	CG
	Adjusted mean change [95% CI]	Adjusted mean change [95% CI]
Short Form-36		
Short Form-36 PCS	−2.9 [−4.2 to −1.6]	−3.4 [−4.8 to −2.1]
M12	−4.1 [−6.0 to −2.3]	−3.6 [−5.6 to −1.7]
M24	−2.6 [−4.6 to −0.5]	−4.4 [−6.5 to −2.2]
Short Form-36 MCS	−0.9 [−2.6 to 0.7]	−0.3 [−2.0 to 1.4]
M12	−0.5 [−2.8 to 1.7]	−1.0 [−3.3 to 1.4]
M24	−0.3 [−2.8 to 2.2]	0.5 [−2.1 to 3.1]
Hospital Anxiety Depression Scale		
Anxiety	−0.3 [−0.9 to 0.2]	−0.2 [−0.7 to 0.4]
M12	−0.3 [−1.0 to 0.5]	0.3 [−0.5 to 1.1]
M24	−0.4 [−1.2 to 0.4]	−0.3 [−1.1 to 0.6]
Depression	0.8 [0.2 to 1.4]	1.1 [0.5 to 1.8]
M12	0.6 [−0.2 to 1.4]	1.3 [0.5 to 2.1]
M24	1.3 [0.4 to 2.1]	**3.4 [0.6 to 2.5]**
Zarit Burden Interview	1.2 [−1.2 to 3.6]	5.2 [2.8 to 7.6]
M12	2.0 [−0.9 to 5.0]	5.7 [2.7 to 8.7]
M24	1.4 [−1.8 to 4.6]	6.4 [3.0 to 9.7]

Note: SIG, Supportive intervention group; CG, control group; M12, M24, Month 12, 24; PCS, physical component summary; MCS, mental component summary; CI, Confidence interval. MID was fixed at 5 points for Short Form-36 PCS and MCS scores, 1.32 points for Hospital Anxiety Depression Scale anxiety score, 1.4 points for Hospital Anxiety Depression Scale depression score, and 7.2 points for Zarit Burden Interview score. In bold: significant MID.

Mixed models for repeated measures used for longitudinal analysis included all timepoints until M24 and included the following effects: randomization group, time, allocation-by-time interaction, adjusted on baseline score, and baseline score-by-time interaction.

### Exploratory Analyses

Exploratory analyses showed several significant changes in caregiver involvement in medical support at M12, in financial assistance (M12, M24), and in medical decision support at M24 in the SIG. In the CG, the rates of caregivers providing help in medical decision support at M12, and in moral and emotional support at M12 and at M24 were significantly reduced compared to baseline. The rate of caregivers requesting professional and financial support was low, regardless of the allocation group. Only a slight increase in professional support was requested at M12 and M24 in the intervention group (data not shown).

## Discussion

The present analysis of 179 caregivers showed no clinically relevant changes in QoL summary scores (SF-36-PCS and -MCS), nor in HADS anxiety and ZBI burden scores, regardless of the allocation group. HADS depression scores significantly increased at M12 and M24 exclusively in the control group, and the mixed models confirmed the mean change results in the control group at M24 (adjusted mean change: 3.4 [0.6–2.5]). No direct comparisons between groups were allowed based on the current sample size.

The deterioration in QoL physical sub-dimensions reported by caregivers in the first 2 years of the ICE study is consistent with previously reported results [32, 33]. Correlation between caregiving stress and physical impairments after 2 years was also previously underlined [32].

In the SIG, no significant changes in depression, and less QoL sub-dimensions with significant deterioration were reported, indirectly reflecting a benefit from social worker intervention. Previous studies investigating intervention in caregivers of patients with dementia also reported quite limited but significant decrease in caregiver depression and burden [34]. Similarly, interventions targeting self-care and interpersonal connections of caregivers, as well as symptom management in adult cancer patients alleviated depression and improved caregiver QoL [35]. The additional support in the SIG contributed to minimizing depression, which is consistent with lower depression scores observed in caregivers with more social relationships as reported by Stenberg et al. [33]. However, in contrast to results from Stenberg et al., our study did not evidence reductions in anxiety and burden.

Caregivers play a critical role and assume in turn several tasks in disease management of patients with cancer [36]. Earlier and better recognition of caregiver involvement in patient care needs to be encouraged. Further issues had arisen regarding the growing recognition of specific caregiver needs, and the most appropriate assistance to offer. Hence, compromised caregiver QoL will also adversely influence the delivery of effective patient care [37].

Clinicians underlined difficulties to appropriately identify the primary caregiver [38]. This finding may contribute to explaining the limited enrolment of caregivers in the ICE study. Caregivers have previously been qualified as “the invisible patient” [39]. The ICE study enrolled nearly 200 caregivers, and although this sample size—far below the pre-planned recruitment—did not allow comparisons in caregivers per patient disease and according to allocation groups, this prospective study gathered a substantial number of caregivers, favorably comparing with previous studies, and pointed to difficulties in reaching this specific population that still need to be overcome [40, 41]. The collaborative efforts to improve access to this informative mixed population of caregivers involved in the support of older patients with different diseases need to be emphasized. Better understanding of the working methods of social workers and improved coordination between social and health systems could help to identify and reach the caregivers more effectively. Further studies specifying caregivers needs, considering the initial pathology of their loved one, and assessing need evolution and changes according to each disease trajectory would be required. A strength of the ICE study is to clearly underline the increased depression over time in the control group, confirmed with the mixed model. This result is consistent with previous reports [32, 33, 42]. Moreover, the informative approach provided by the longitudinal follow-up of 2 years needs to be highlighted. However, the global interpretation of the changes in QoL should be cautious because of attrition biases. Indeed, caregivers having discontinued the study early were the most at risk for limited QoL with poor physical dimensions and high burden at baseline in caregivers withdrawn at M12. To what extent social support contributes to alleviating depression needs to be further investigated.

To date, no specific assessment of living conditions, including accurate identification of caregiving is performed at the initiation of patient management, and conducting research on caregivers remains a challenging issue [43], and other initiatives either considering patient-caregiver dyads, or family-centered approaches should also be encouraged and integrated in further clinical studies and in patient care. A framework to address the needs of caregivers has been recently proposed in early palliative care in cancer [12]. Further evidence-based studies with interventions from social workers, nurses, support groups, or integrated in early palliative care, and collaborations thereof are promising alternatives which need to be further explored [11–13].

The innovative and ambitious ICE program received funding from the National research agency and the French National Institute of Cancer and aimed to raise awareness and recognition of the role caregiver, improve self-recognition and support, and consider caregivers as part of the unit of care. Along with large public information campaigns in the last years, incentives to strengthen the relationship between health and humanities and social sciences structures are encouraged. The ICE study focused on the support to be delivered to the still difficult to reach population of caregivers, on an individual level. The study provided useful insights into caregiver characterization. While caregiver depression increased over time in this population, social worker intervention proved promising. These results underlined the critical need to better support caregivers of older patients and urge support to be adjusted with adequate methods, resources, and timing elements. Indeed, social workers reported that they mainly had a role of listening and returned that caregivers had not taken advantage of the full scope of opportunities they may provide. Several assumptions may explain such missed opportunities. The protocol scheduled semester visits that did not correspond to the current social worker practice, usually proposing more closely spaced visits. This may have contributed to limiting the ability of social workers to provide appropriate timely support. More frequent visits, organized at the request of caregivers, would have encouraged the emergence of needs; therefore, caregiver needs could have been more appropriately and efficiently addressed.

The term “informal” is largely used worldwide to qualify unpaid caregiving. However, this term may still lack conceptual clarity, and controversial views have been reported highlighting the negative connotation that “Informal” may include, faced with the substantial efforts in supporting and directly caring for patients over a long period of time that the caregivers, identified or not, supported or not, currently provide.

Health policy developments and local regulation have already evolved in recent years and have been recognized as essential to provide adequate support to this vulnerable population and new institutional structures have been created to alleviate caregiver burden in France [44], and other initiatives are currently being tested worldwide [45, 46]. Providing caregivers with an opportunity to rest and recover is essential for maintaining their capacity to care for their loved ones, and greater recognition of needs, issues, and rights during disease course is required. Caregiver respite and emotional needs are of particular importance at specific periods/timepoints, notably in assisting cancer patients in the first 12 months after diagnosis [47].

### Conclusion

A global approach strengthening collaborations between social and health systems proved promising. Social support may contribute to preventing or reducing caregiver depression at 1 and 2 years and therefore prevent deterioration in global caregiver QoL. If randomized studies are needed to further define and investigate personalized reliable interventions, a greater use of the large currently available expertise of social workers, supported by already existing nationwide networks is also a contributory promising approach. This social involvement would allow to better reach the caregivers, in a complementary and consistent manner, to provide better identification and characterization of caregivers, and a better understanding of caregiver needs, especially early in the course of the patient disease. Timely organization of dedicated scalable support to the caregivers the most in need could be implemented. Tailored interventions from all available sources, including help provided by the social system, are required to adequately support this important, vulnerable, and still too invisible population.

## Data Availability

The datasets analysed during the current study are available from the corresponding author on reasonable request.
