# Supplementary material for: Quality of Life and Needs in Caregivers: Results From the Prospective Multicentric Open-Label Randomized Study of Informal Caregivers of Elderly Patients
Source: Int J Public Health. 2023 Aug 30;68:1605459. doi: 10.3389/ijph.2023.1605459 (PMC10498993; doi:10.3389/ijph.2023.1605459)
Supplement: Supplementary file 1 [file Table1.docx]

**SUPPLEMENTARY MATERIAL**

**Table S1. Attrition at Month 12 and at Month 24. Baseline characteristics between caregivers remaining in the study and caregivers who discontinued at A) Month 12; B) Month 24 (Informal Carers of Elderly study, France, 2015-2019).**

A)

|  | Caregivers having discontinued before Month 12 | | | | All | | Test |
| --- | --- | --- | --- | --- | --- | --- | --- |
|  | No | | Yes | |  |  |  |
|  | N=152 | | N=27 | | N=179 | |  |
| **Randomisation group** |  |  |  |  |  |  |  |
| Control group | 77 | (50.7%) | 12 | (44.4%) | 89 | (49.7%) |  |
| Supportive intervention group | 75 | (49.3%) | 15 | (55.6%) | 90 | (50.3%) |  |
| **Patient characteristics** |  |  |  |  |  |  |  |
| Gender |  |  |  |  |  |  | Chi-2    P = 0.100 |
| Male | 71 | (46.7%) | 8 | (29.6%) | 79 | (44.1%) |  |
| Female | 81 | (53.3%) | 19 | (70.4%) | 100 | (55.9%) |  |
| Age |  |  |  |  |  |  | T-test    P = 0.529 |
| N | 152 | | 27 | | 179 | |  |
| Median (min-max) | 72.0 (60-94) | | 74.0 (61-93) | | 73.0 (60-94) | |  |
| Disease (recently diagnosed) |  |  |  |  |  |  | Chi-2    P = 0.524 |
| Alzheimer and related diseases | 39 | (25.7%) | 7 | (25.9%) | 46 | (25.7%) |  |
| Parkinson | 16 | (10.5%) | 4 | (14.8%) | 20 | (11.2%) |  |
| Stroke | 9 | (5.9%) | 1 | (3.7%) | 10 | (5.6%) |  |
| AMD | 9 | (5.9%) | 2 | (7.4%) | 11 | (6.1%) |  |
| Colorectal cancer | 19 | (12.5%) | 2 | (7.4%) | 21 | (11.7%) |  |
| Prostate cancer | 24 | (15.8%) | 1 | (3.7%) | 25 | (14.0%) |  |
| Breast cancer | 36 | (23.7%) | 10 | (37.0%) | 46 | (25.7%) |  |
| **Caregiver characteristics** |  |  |  |  |  |  |  |
| Gender |  |  |  |  |  |  | Chi-2    P = 0.068 |
| Male | 46 | (30.3%) | 13 | (48.1%) | 59 | (33.0%) |  |
| Female | 106 | (69.7%) | 14 | (51.9%) | 120 | (67.0%) |  |
| Age |  |  |  |  |  |  | T-test    P = 0.993 |
| N | 152 | | 27 | | 179 | |  |
| Median (min-max) | 65.0 (29-92) | | 67.0 (36-88) | | 65.0 (29-92) | |  |
| Marital status / living situation |  |  |  |  |  |  | Chi-2    P = 0.832 |
| Married, common-law couple, couple | 127 | (83.6%) | 23 | (85.2%) | 150 | (83.8%) |  |
| Other (single, separated, divorced or widowed) | 25 | (16.4%) | 4 | (14.8%) | 29 | (16.2%) |  |
| Caregiver-patient relationship |  |  |  |  |  |  | Chi-2    P = 0.935 |
| Spouse | 101 | (66.4%) | 17 | (63.0%) | 118 | (65.9%) |  |
| Mother/Father | 35 | (23.0%) | 7 | (25.9%) | 42 | (23.5%) |  |
| Other family members (sister, brother, mother/father-in-law, uncle/aunt, grand-mother) | 8 | (5.3%) | 2 | (7.4%) | 10 | (5.6%) |  |
| Other (friend, neighbour, ex-husband) | 8 | (5.3%) | 1 | (3.7%) | 9 | (5.0%) |  |
| Professional situation |  |  |  |  |  |  | Chi-2    P = 0.790 |
| Professional activity | 37 | (24.3%) | 5 | (18.5%) | 42 | (23.5%) |  |
| Retired | 103 | (67.8%) | 20 | (74.1%) | 123 | (68.7%) |  |
| Other | 12 | (7.9%) | 2 | (7.4%) | 14 | (7.8%) |  |
| Household incomes €/month |  |  |  |  |  |  | Chi-2    P = 0.975 |
| Missing | 19 |  | 1 |  | 20 |  |  |
| < €800 | 5 | (3.8%) | 1 | (3.8%) | 6 | (3.8%) |  |
| From €800 to €1,500 | 14 | (10.5%) | 3 | (11.5%) | 17 | (10.7%) |  |
| From €1,501 to €3,000 | 73 | (54.9%) | 13 | (50.0%) | 86 | (54.1%) |  |
| > €3,000 | 41 | (30.8%) | 9 | (34.6%) | 50 | (31.4%) |  |
| Help requested by the caregiver |  |  |  |  |  |  |  |
| Financial help requested by the caregiver |  |  |  |  |  |  | Chi-2    P = 0.755 |
| No | 148 | (97.4%) | 26 | (96.3%) | 174 | (97.2%) |  |
| Yes | 4 | (2.6%) | 1 | (3.7%) | 5 | (2.8%) |  |
| Professional help requested by the caregiver |  |  |  |  |  |  | Chi-2    P = 0.890 |
| No | 131 | (86.2%) | 23 | (85.2%) | 154 | (86.0%) |  |
| Yes | 21 | (13.8%) | 4 | (14.8%) | 25 | (14.0%) |  |
| Involvement in patient activities |  |  |  |  |  |  |  |
| Daily living activities |  |  |  |  |  |  | Chi-2    P = 0.372 |
| No | 138 | (90.8%) | 23 | (85.2%) | 161 | (89.9%) |  |
| Yes | 14 | (9.2%) | 4 | (14.8%) | 18 | (10.1%) |  |
| Domestic chores |  |  |  |  |  |  | Chi-2    P = 0.399 |
| No | 52 | (34.2%) | 7 | (25.9%) | 59 | (33.0%) |  |
| Yes | 100 | (65.8%) | 20 | (74.1%) | 120 | (67.0%) |  |
| Administrative management |  |  |  |  |  |  | Chi-2    P = 0.364 |
| No | 53 | (34.9%) | 7 | (25.9%) | 60 | (33.5%) |  |
| Yes | 99 | (65.1%) | 20 | (74.1%) | 119 | (66.5%) |  |
| Medical support |  |  |  |  |  |  | Chi-2    P = 0.468 |
| No | 38 | (25.0%) | 5 | (18.5%) | 43 | (24.0%) |  |
| Yes | 114 | (75.0%) | 22 | (81.5%) | 136 | (76.0%) |  |
| Physical support services |  |  |  |  |  |  | Chi-2    P = 0.484 |
| No | 106 | (69.7%) | 17 | (63.0%) | 123 | (68.7%) |  |
| Yes | 46 | (30.3%) | 10 | (37.0%) | 56 | (31.3%) |  |
| Financial assistance |  |  |  |  |  |  | Chi-2    P = 0.238 |
| No | 75 | (49.3%) | 10 | (37.0%) | 85 | (47.5%) |  |
| Yes | 77 | (50.7%) | 17 | (63.0%) | 94 | (52.5%) |  |
| Moral and emotional support |  |  |  |  |  |  | Chi-2    P = 0.952 |
| No | 6 | (3.9%) | 1 | (3.7%) | 7 | (3.9%) |  |
| Yes | 146 | (96.1%) | 26 | (96.3%) | 172 | (96.1%) |  |
| Medical decision support |  |  |  |  |  |  | Chi-2    P = 0.212 |
| No | 29 | (19.1%) | 8 | (29.6%) | 37 | (20.7%) |  |
| Yes | 123 | (80.9%) | 19 | (70.4%) | 142 | (79.3%) |  |
| **Short Form-36 scores** |  |  |  |  |  |  |  |
| Physical Functioning |  |  |  |  |  |  | T-test    P = 0.282 |
| N | 152 | | 27 | | 179 | |  |
| Mean (SD) | 86.4 (18.6) | | 82.2 (17.2) | | 85.7 (18.4) | |  |
| Median (min-max) | 92.5 (5-100) | | 85.0 (40-100) | | 90.0 (5-100) | |  |
| Role Physical |  |  |  |  |  |  | T-test    P = 0.028 |
| N | 152 | | 27 | | 179 | |  |
| Mean (SD) | 74.8 (36.3) | | 57.4 (44.3) | | 72.2 (38.0) | |  |
| Median (min-max) | 100.0 (0-100) | | 75.0 (0-100) | | 100.0 (0-100) | |  |
| Bodily Pain |  |  |  |  |  |  | T-test    P = 0.369 |
| N | 152 | | 27 | | 179 | |  |
| Mean (SD) | 61.9 (33.2) | | 55.6 (37.5) | | 61.0 (33.8) | |  |
| Median (min-max) | 74.0 (0-100) | | 40.0 (0-100) | | 74.0 (0-100) | |  |
| Mental Health |  |  |  |  |  |  | T-test    P = 0.254 |
| N | 152 | | 27 | | 179 | |  |
| Mean (SD) | 63.5 (18.9) | | 59.0 (19.6) | | 62.8 (19.0) | |  |
| Median (min-max) | 66.0 (12-100) | | 56.0 (20-88) | | 64.0 (12-100) | |  |
| Role Emotional |  |  |  |  |  |  | T-test    P = 0.095 |
| N | 152 | | 27 | | 179 | |  |
| Mean (SD) | 70.6 (38.2) | | 56.8 (46.1) | | 68.5 (39.6) | |  |
| Median (min-max) | 100.0 (0-100) | | 66.7 (0-100) | | 100.0 (0-100) | |  |
| Social Functioning |  |  |  |  |  |  | T-test    P = 0.171 |
| N | 152 | | 27 | | 179 | |  |
| Mean (SD) | 79.3 (20.4) | | 73.1 (26.3) | | 78.4 (21.4) | |  |
| Median (min-max) | 87.5 (25-100) | | 75.0 (13-100) | | 87.5 (13-100) | |  |
| Vitality |  |  |  |  |  |  | T-test    P = 0.035 |
| N | 152 | | 27 | | 179 | |  |
| Mean (SD) | 58.7 (19.0) | | 50.2 (19.8) | | 57.4 (19.3) | |  |
| Median (min-max) | 60.0 (15-100) | | 45.0 (15-95) | | 55.0 (15-100) | |  |
| General Health |  |  |  |  |  |  | T-test    P = 0.547 |
| N | 152 | | 27 | | 179 | |  |
| Mean (SD) | 63.7 (17.3) | | 61.5 (20.4) | | 63.4 (17.7) | |  |
| Median (min-max) | 67.0 (10-100) | | 72.0 (25-87) | | 67.0 (10-100) | |  |
| Health Transition |  |  |  |  |  |  | T-test    P = 0.025 |
| N | 152 | | 27 | | 179 | |  |
| Mean (SD) | 50.3 (17.0) | | 42.6 (11.6) | | 49.2 (16.5) | |  |
| Median (min-max) | 50.0 (25-100) | | 50.0 (25-50) | | 50.0 (25-100) | |  |
| **Physical Component Summary** |  |  |  |  |  |  | T-test    P = 0.186 |
| N | 152 | | 27 | | 179 | |  |
| Mean (SD) | 48.8 (8.8) | | 46.3 (9.1) | | 48.4 (8.8) | |  |
| Median (min-max) | 50.2 (23-64) | | 47.6 (29-58) | | 50.0 (23-64) | |  |
| **Mental Component Summary** |  |  |  |  |  |  | T-test    P = 0.126 |
| N | 152 | | 27 | | 179 | |  |
| Mean (SD) | 45.4 (10.4) | | 42.0 (12.0) | | 44.9 (10.6) | |  |
| Median (min-max) | 47.2 (15-64) | | 46.3 (22-59) | | 47.2 (15-64) | |  |
| **Hospital Anxiety Depression Scale anxiety and depression scores** |  |  |  |  |  |  |  |
| Anxiety |  |  |  |  |  |  | T-test    P = 0.374 |
| N | 152 | | 27 | | 179 | |  |
| Mean (SD) | 7.7 (3.9) | | 8.4 (5.2) | | 7.8 (4.2) | |  |
| Median (min-max) | 7.0 (1-21) | | 8.0 (1-20) | | 7.0 (1-21) | |  |
| Depression |  |  |  |  |  |  | T-test    P = 0.112 |
| N | 152 | | 27 | | 179 | |  |
| Mean (SD) | 4.2 (3.5) | | 5.3 (3.6) | | 4.3 (3.5) | |  |
| Median (min-max) | 3.0 (0-14) | | 4.0 (0-13) | | 4.0 (0-14) | |  |
| **Zarit burden Interview** |  |  |  |  |  |  |  |
| Zarit burden score |  |  |  |  |  |  | T-test    P = 0.035 |
| N | 149 | | 26 | | 175 | |  |
| Mean (SD) | 17.8 (13.7) | | 24.2 (17.4) | | 18.7 (14.4) | |  |
| Median (min-max) | 14.0 (0-60) | | 20.0 (3-62) | | 15.0 (0-62) | |  |

B)

|  | Caregivers having discontinued before Month 24 | | | | All | | Test |
| --- | --- | --- | --- | --- | --- | --- | --- |
|  | No | | Yes | |  |  |  |
|  | N=113 | | N=66 | | N=179 | |  |
| **Randomisation group** |  |  |  |  |  |  |  |
|  |  |  |  |  |  |  | Chi-2    P = 0.800 |
| Control group | 57 | (50.4%) | 32 | (48.5%) | 89 | (49.7%) |  |
| Supportive intervention group | 56 | (49.6%) | 34 | (51.5%) | 90 | (50.3%) |  |
| **Patient characteristics** |  |  |  |  |  |  |  |
| Gender |  |  |  |  |  |  | Chi-2    P = 0.725 |
| Male | 51 | (45.1%) | 28 | (42.4%) | 79 | (44.1%) |  |
| Female | 62 | (54.9%) | 38 | (57.6%) | 100 | (55.9%) |  |
| Age |  |  |  |  |  |  | T-test    P = 0.636 |
| N | 113 | | 66 | | 179 | |  |
| Median (min-max) | 72.0 (60-94) | | 73.5 (60-94) | | 73.0 (60-94) | |  |
| Disease recently diagnosed |  |  |  |  |  |  | Chi-2    P = 0.261 |
| Alzheimer and related disease | 27 | (23.9%) | 19 | (28.8%) | 46 | (25.7%) |  |
| Parkinson | 12 | (10.6%) | 8 | (12.1%) | 20 | (11.2%) |  |
| Stroke | 8 | (7.1%) | 2 | (3.0%) | 10 | (5.6%) |  |
| AMD | 8 | (7.1%) | 3 | (4.5%) | 11 | (6.1%) |  |
| Colo-rectal cancer | 9 | (8.0%) | 12 | (18.2%) | 21 | (11.7%) |  |
| Prostate cancer | 19 | (16.8%) | 6 | (9.1%) | 25 | (14.0%) |  |
| Breast cancer | 30 | (26.5%) | 16 | (24.2%) | 46 | (25.7%) |  |
| **Caregiver characteristics** |  |  |  |  |  |  |  |
| Gender |  |  |  |  |  |  | Chi-2    P = 0.681 |
| Male | 36 | (31.9%) | 23 | (34.8%) | 59 | (33.0%) |  |
| Female | 77 | (68.1%) | 43 | (65.2%) | 120 | (67.0%) |  |
| Age |  |  |  |  |  |  | T-test    P = 0.862 |
| N | 113 | | 66 | | 179 | |  |
| Median (min-max) | 66.0 (29-88) | | 65.0 (36-92) | | 65.0 (29-92) | |  |
| Marital status / living situation |  |  |  |  |  |  | Chi-2    P = 0.332 |
| Married, common-law couple, couple | 97 | (85.8%) | 53 | (80.3%) | 150 | (83.8%) |  |
| Other (single, separated, divorced or widowed) | 16 | (14.2%) | 13 | (19.7%) | 29 | (16.2%) |  |
| Caregiver-patient relationship |  |  |  |  |  |  | Chi-2    P = 0.286 |
| Spouse | 76 | (67.3%) | 42 | (63.6%) | 118 | (65.9%) |  |
| Mother/Father | 28 | (24.8%) | 14 | (21.2%) | 42 | (23.5%) |  |
| Other family members (sister, brother,mother/father-in-law, uncle/aunt, grand-mother) | 6 | (5.3%) | 4 | (6.1%) | 10 | (5.6%) |  |
| Other (friend, neighbour, ex-husband) | 3 | (2.7%) | 6 | (9.1%) | 9 | (5.0%) |  |
| Professional situation |  |  |  |  |  |  | Chi-2    P = 0.470 |
| Professional activity | 29 | (25.7%) | 13 | (19.7%) | 42 | (23.5%) |  |
| Retired | 74 | (65.5%) | 49 | (74.2%) | 123 | (68.7%) |  |
| Other | 10 | (8.8%) | 4 | (6.1%) | 14 | (7.8%) |  |
| Household incomes €/month |  |  |  |  |  |  | Chi-2    P = 0.862 |
| Missing | 13 |  | 7 |  | 20 |  |  |
| < €800 | 3 | (3.0%) | 3 | (5.1%) | 6 | (3.8%) |  |
| From €800 to €1,500 | 10 | (10.0%) | 7 | (11.9%) | 17 | (10.7%) |  |
| From €1,501 to €3,000 | 56 | (56.0%) | 30 | (50.8%) | 86 | (54.1%) |  |
| > €3,000 | 31 | (31.0%) | 19 | (32.2%) | 50 | (31.4%) |  |
| **Help requested by the caregiver** |  |  |  |  |  |  |  |
| Financial help requested by the caregiver |  |  |  |  |  |  | Chi-2    P = 0.277 |
| No | 111 | (98.2%) | 63 | (95.5%) | 174 | (97.2%) |  |
| Yes | 2 | (1.8%) | 3 | (4.5%) | 5 | (2.8%) |  |
| Professional help requested by the caregiver |  |  |  |  |  |  | Chi-2    P = 0.033 |
| No | 102 | (90.3%) | 52 | (78.8%) | 154 | (86.0%) |  |
| Yes | 11 | (9.7%) | 14 | (21.2%) | 25 | (14.0%) |  |
| **Involvement in patient activities** |  |  |  |  |  |  |  |
| Daily living activities |  |  |  |  |  |  | Chi-2    P = 0.083 |
| No | 105 | (92.9%) | 56 | (84.8%) | 161 | (89.9%) |  |
| Yes | 8 | (7.1%) | 10 | (15.2%) | 18 | (10.1%) |  |
| Domestic chores |  |  |  |  |  |  | Chi-2    P = 0.117 |
| No | 42 | (37.2%) | 17 | (25.8%) | 59 | (33.0%) |  |
| Yes | 71 | (62.8%) | 49 | (74.2%) | 120 | (67.0%) |  |
| Administrative management |  |  |  |  |  |  | Chi-2    P = 0.486 |
| No | 40 | (35.4%) | 20 | (30.3%) | 60 | (33.5%) |  |
| Yes | 73 | (64.6%) | 46 | (69.7%) | 119 | (66.5%) |  |
| Medical support |  |  |  |  |  |  | Chi-2    P = 0.013 |
| No | 34 | (30.1%) | 9 | (13.6%) | 43 | (24.0%) |  |
| Yes | 79 | (69.9%) | 57 | (86.4%) | 136 | (76.0%) |  |
| Physical support services |  |  |  |  |  |  | Chi-2    P = 0.146 |
| No | 82 | (72.6%) | 41 | (62.1%) | 123 | (68.7%) |  |
| Yes | 31 | (27.4%) | 25 | (37.9%) | 56 | (31.3%) |  |
| Financial assistance |  |  |  |  |  |  | Chi-2    P = 0.468 |
| No | 56 | (49.6%) | 29 | (43.9%) | 85 | (47.5%) |  |
| Yes | 57 | (50.4%) | 37 | (56.1%) | 94 | (52.5%) |  |
| Moral and emotional support |  |  |  |  |  |  | Chi-2    P = 0.206 |
| No | 6 | (5.3%) | 1 | (1.5%) | 7 | (3.9%) |  |
| Yes | 107 | (94.7%) | 65 | (98.5%) | 172 | (96.1%) |  |
| Medical decision support |  |  |  |  |  |  | Chi-2    P = 0.806 |
| No | 24 | (21.2%) | 13 | (19.7%) | 37 | (20.7%) |  |
| Yes | 89 | (78.8%) | 53 | (80.3%) | 142 | (79.3%) |  |
| **Short Form-36 scores** |  |  |  |  |  |  |  |
| Physical Functioning |  |  |  |  |  |  | T-test    P = 0.018 |
| N | 113 | | 66 | | 179 | |  |
| Mean (SD) | 88.2 (15.1) | | 81.5 (22.6) | | 85.7 (18.4) | |  |
| Median (min-max) | 95.0 (30-100) | | 87.5 (5-100) | | 90.0 (5-100) | |  |
| Role Physical |  |  |  |  |  |  | T-test    P = 0.112 |
| N | 113 | | 66 | | 179 | |  |
| Mean (SD) | 75.7 (34.6) | | 66.3 (42.9) | | 72.2 (38.0) | |  |
| Median (min-max) | 100.0 (0-100) | | 100.0 (0-100) | | 100.0 (0-100) | |  |
| Bodily Pain |  |  |  |  |  |  | T-test    P = 0.348 |
| N | 113 | | 66 | | 179 | |  |
| Mean (SD) | 62.8 (32.4) | | 57.8 (36.2) | | 61.0 (33.8) | |  |
| Median (min-max) | 74.0 (10-100) | | 74.0 (0-100) | | 74.0 (0-100) | |  |
| Mental Health |  |  |  |  |  |  | T-test    P = 0.641 |
| N | 113 | | 66 | | 179 | |  |
| Mean (SD) | 63.3 (19.5) | | 62.0 (18.4) | | 62.8 (19.0) | |  |
| Median (min-max) | 68.0 (12-100) | | 60.0 (20-92) | | 64.0 (12-100) | |  |
| Role Emotional |  |  |  |  |  |  | T-test    P = 0.543 |
| N | 113 | | 66 | | 179 | |  |
| Mean (SD) | 69.9 (37.0) | | 66.2 (43.9) | | 68.5 (39.6) | |  |
| Median (min-max) | 100.0 (0-100) | | 100.0 (0-100) | | 100.0 (0-100) | |  |
| Social Functioning |  |  |  |  |  |  | T-test    P = 0.291 |
| N | 113 | | 66 | | 179 | |  |
| Mean (SD) | 79.6 (20.7) | | 76.1 (22.4) | | 78.4 (21.4) | |  |
| Median (min-max) | 87.5 (25-100) | | 81.3 (13-100) | | 87.5 (13-100) | |  |
| Vitality |  |  |  |  |  |  | T-test    P = 0.114 |
| N | 113 | | 66 | | 179 | |  |
| Mean (SD) | 59.1 (18.9) | | 54.4 (19.8) | | 57.4 (19.3) | |  |
| Median (min-max) | 60.0 (15-100) | | 52.5 (15-100) | | 55.0 (15-100) | |  |
| General Health |  |  |  |  |  |  | T-test    P = 0.276 |
| N | 113 | | 66 | | 179 | |  |
| Mean (SD) | 64.5 (16.4) | | 61.5 (19.7) | | 63.4 (17.7) | |  |
| Median (min-max) | 67.0 (27-100) | | 62.3 (10-100) | | 67.0 (10-100) | |  |
| Health Transition |  |  |  |  |  |  | T-test    P = 0.676 |
| N | 113 | | 66 | | 179 | |  |
| Mean (SD) | 49.6 (16.4) | | 48.5 (16.9) | | 49.2 (16.5) | |  |
| Median (min-max) | 50.0 (25-100) | | 50.0 (25-100) | | 50.0 (25-100) | |  |
| **Physical Component Summary** |  |  |  |  |  |  | T-test    P = 0.033 |
| N | 113 | | 66 | | 179 | |  |
| Mean (SD) | 49.5 (8.1) | | 46.6 (9.9) | | 48.4 (8.8) | |  |
| Median (min-max) | 50.7 (28-64) | | 48.2 (23-63) | | 50.0 (23-64) | |  |
| **Mental Component Summary** |  |  |  |  |  |  | T-test    P = 0.727 |
| N | 113 | | 66 | | 179 | |  |
| Mean (SD) | 45.1 (10.7) | | 44.5 (10.6) | | 44.9 (10.6) | |  |
| Median (min-max) | 47.7 (15-63) | | 46.5 (22-64) | | 47.2 (15-64) | |  |
| **Hospital Anxiety DepressionScale anxiety and depression scores** |  |  |  |  |  |  |  |
| Anxiety |  |  |  |  |  |  | T-test    P = 0.824 |
| N | 113 | | 66 | | 179 | |  |
| Mean (SD) | 7.8 (4.0) | | 7.7 (4.5) | | 7.8 (4.2) | |  |
| Median (min-max) | 7.0 (2-21) | | 7.0 (1-20) | | 7.0 (1-21) | |  |
| Depression |  |  |  |  |  |  | T-test    P = 0.055 |
| N | 113 | | 66 | | 179 | |  |
| Mean (SD) | 4.0 (3.4) | | 5.0 (3.6) | | 4.3 (3.5) | |  |
| Median (min-max) | 3.0 (0-14) | | 4.0 (0-13) | | 4.0 (0-14) | |  |
| **Zarit burden Interview** |  |  |  |  |  |  |  |
| Zarit burden score |  |  |  |  |  |  | T-test    P = 0.177 |
| N | 111 | | 64 | | 175 | |  |
| Mean (SD) | 17.6 (14.3) | | 20.7 (14.6) | | 18.7 (14.4) | |  |
| Median (min-max) | 14.0 (0-60) | | 19.0 (0-62) | | 15.0 (0-62) | |  |

Note: Daily living activities (grooming/dressing, etc); Domestic chores (cleaning, grocery shopping, meals, etc); Administrative management (accounting, mails, decisions); Medical support (accompaniment to medical appointments, medical cares). AMD: Age-related macular degeneration.

At Month 12, in comparison to caregivers who remained in the study, caregivers who early discontinued the study presented at baseline lower mean scores in Short Form-36 role physical (57.4 ±44.3 vs 74.8 ±36.3), vitality score (50.2 ±19.8 vs 58.7 ±19.0), health transition (42.6 ±11.6 vs 50.3 ±17.0) and a higher Zarit Burden Interview score (24.2 ±17.4 vs 17.8 ±13.7).

At Month 24, in comparison to caregivers still in the study, caregivers who early discontinued of the study requested more professional help (21% vs 10%), provided more medical support to the patient (86% vs 70%), and presented at baseline a lower Short Form-36 physical functioning score (mean 81.5 ±22.6 vs 88.2 ±15.1) and Physical Component Summary (46.6 ±9.9 vs 49.5 ±8.1).
